# Improving gastric cancer preclinical studies using diverse in vitro and in vivo model systems

**DOI:** 10.1186/s12885-016-2232-2

**Published:** 2016-03-09

**Authors:** Hae Ryung Chang, Hee Seo Park, Young Zoo Ahn, Seungyoon Nam, Hae Rim Jung, Sungjin Park, Sang Jin Lee, Curt Balch, Garth Powis, Ja-Lok Ku, Yon Hui Kim

**Affiliations:** New Experimental Therapeutics Branch, National Cancer Center of Korea, Ilsan, Goyang-si, Gyeonggi-do Republic of Korea; Cancer Biology Research Laboratory, Institut Pasteur Korea, Bundang, Seongnam-si, Gyeonggi-do Republic of Korea; Animal Sciences Branch, National Cancer Center of Korea, Ilsan, Goyang-si, Gyeonggi-do Republic of Korea; Department of Life Sciences, College of BioNano Technology, Gachon University, Sungnam, South Korea; College of Medicine, Gachon University, Incheon, South Korea; Department of Pharmacology and Experimental Therapeutics, University of Toledo College of Pharmacy, Toledo, OH USA; Cancer Center, Sanford-Burnham-Prebys Medical Discovery Institute, La Jolla, CA USA; SNU Korean Cell Line Bank, Cancer Research Institute, Seoul National University, Seoul, Republic of Korea

**Keywords:** Biomarker, Cell microarray, ERBB2 expression, Gastric cancer cell lines, Targeted therapies, Trastuzumab, Tumor heterogeneity, Xenograft microarray

## Abstract

**Background:**

“Biomarker-driven targeted therapy,” the practice of tailoring patients’ treatment to the expression/activity levels of disease-specific genes/proteins, remains challenging. For example, while the anti-ERBB2 monoclonal antibody, trastuzumab, was first developed using well-characterized, diverse in vitro breast cancer models (and is now a standard adjuvant therapy for ERBB2-positive breast cancer patients), trastuzumab approval for ERBB2-positive gastric cancer was largely based on preclinical studies of a single cell line, NCI-N87. Ensuing clinical trials revealed only modest patient efficacy, and many ERBB2-positive gastric cancer (GC) patients failed to respond at all (i.e., were inherently recalcitrant), or succumbed to acquired resistance.

**Method:**

To assess mechanisms underlying GC insensitivity to ERBB2 therapies, we established a diverse panel of GC cells, differing in ERBB2 expression levels, for comprehensive in vitro and in vivo characterization. For higher throughput assays of ERBB2 DNA and protein levels, we compared the concordance of various laboratory quantification methods, including those of in vitro and in vivo genetic anomalies (FISH and SISH) and xenograft protein expression (Western blot vs. IHC), of both cell and xenograft (tissue-sectioned) microarrays.

**Results:**

The biomarker assessment methods strongly agreed, as did correlation between RNA and protein expression. However, although *ERBB2* genomic anomalies showed good in vitro vs. in vivo correlation, we observed striking differences in protein expression between cultured cells and mouse xenografts (even within the same GC cell type). Via our unique pathway analysis, we delineated a signaling network, in addition to specific pathways/biological processes, emanating from the ERBB2 signaling cascade, as a potential useful target of clinical treatment. Integrated analysis of public data from gastric tumors revealed frequent (10 – 20 %) amplification of the genes *NFKBIE*, *PTK2*, and *PIK3CA,* each of which resides in an ERBB2-derived subpathway network.

**Conclusion:**

Our comprehensive bioinformatics analyses of highly heterogeneous cancer cells, combined with tumor “omics” profiles, can optimally characterize the expression patterns and activity of specific tumor biomarkers. Subsequent in vitro and in vivo validation, of specific disease biomarkers (using multiple methodologies), can improve prediction of patient stratification according to drug response or nonresponse.

**Electronic supplementary material:**

The online version of this article (doi:10.1186/s12885-016-2232-2) contains supplementary material, which is available to authorized users.

## Background

A promising approach for the treatment of cancer is the use of “targeted” therapies for patients possessing specific genomic anomalies or overexpressing certain oncoproteins, resulting in attenuation of mitogenic signal pathways comprised of such targeted biomolecules [1]. Targeted therapies can avoid the toxicity and eventual drug resistance associated with standard chemo- or radiotherapies [1]. The successful discovery of targeted therapies is based on findings that the majority of cell lines retain the “addictive” driver gene mutations of their originating tumors [2], followed by rigorous in vitro and in vivo preclinical cell line analyses of the candidate therapeutic targets, justifying further progression toward clinical trials [3].

The revolution of targeted therapies, also designated “personalized” (or “precision”) medicine,” holds immense potential, ultimately allowing simple processing of a biopsy to generate massive genomic/transcriptomic data regarding the heterogeneity of a specific tumor [4] active oncogenic pathways, immunoevasive measures employed by circulating cancer cells [5], and resistance mechanisms of drug bypass [6].

While distinct cancer phenotype-associated “signatures,” based on the presence of hundreds (perhaps eventually, even thousands) of expressed/silenced genes, mutation patterns, etc., such considerable assessments have yet to be approved for the clinic [7].

Consequently, patient stratification methods remain largely restricted to single or a few gene/protein biomarkers. Other barriers to successful personalized medicine include inadequate “clinical utility,” referring to knowledge that a biomarker not only statistically segregates two patient populations (“analytical validation”), but that it does so in a clinical meaningful manner (“clinical validation”) [8]. Toward this objective, Hayes et al. assert that an individual biomarker test must be “accurate, reproducible and reliable” and that regulatory bodies have lagged in vetting biomarkers to the same extent as new pharmaceuticals [8]. Consequently, high-quality preclinical studies, using assays relevant to the clinical question at hand, are greatly needed.

Despite these remaining obstacles, several biomarker-based therapies have now been clinically approved, including erlotinib, cetuximab and gefitinib (targeting the epidermal growth factor receptor) [9], bevacizumab (targeting the vascular endothelial growth factor receptor) [10], and imatinib and dasatinib (targeting the bcr-abl translocated tyrosine kinase gene) [11].

However, like conventional drugs, resistance to targeted therapies often arises, due to heterogeneity of expression of the target, additional genomic alterations, or a shift in cancer cell growth reliance to alternative signal pathways (i.e, loss of “addiction” to the targeted pathway) [6, 12]. Consequently, there is an urgent need to identify more reliable biomarkers that predict which patients will best respond to specific targeted compounds vs. those with inherent or predicted acquired resistance. One example of such a therapeutically targeted biomarker is the anti-ERBB2 monoclonal antibody, trastuzumab (Herceptin®, Genentech), developed using multiple, diverse ERBB2-overexpressing breast cancer cell lines [13, 14], which is now a standard adjuvant therapy for ERBB2-positive breast cancer patients [15]. ERBB2, commonly known as HER2 (human epidermal growth factor receptor-2), has no known direct ligand-binding domain, but is a common dimerization partner for the three other EGFR family proteins, stimulating its autophosphorylation activity [16]. Following activation, the ERBB2 intracellular “docking” site interacts with src-homology-2 (SH2) domain proteins, initiating signaling that ultimately results in cell proliferation and the inhibition of cell cycle arrest and apoptosis [17].

While trastuzumab has been well established as successful against ERBB2-positive breast cancer, preclinical studies of the efficacy of trastuzumab against gastric cancer (GC) were largely restricted to a single cell line, NCI-N87, expressing extremely high levels of ERBB2 [18, 19]. Although trastuzumab is now nearly globally approved for GC, the largest phase III clinical trial to date showed only a limited benefit (median overall survival of 13.8 months in those receiving trastuzumab plus chemotherapy, compared to 11.1 months for patients receiving chemotherapy alone) [20]. One could postulate that the difference in trastuzumab clinical efficacy between gastric and breast cancers is correlated to the paucity of preclinical studies of divergent GC cell lines. Similarly, a poor response rate was observed in a phase 1/II trastuzumab clinical trial for ovarian cancer, following limited preclinical studies [21].

In this study, we show wide disparity between in vitro (culture) vs. in vivo (xenograft) ERBB2 protein expression in a panel of 25 diverse GC cell lines, and through our unique subpathway analysis (PATHOME) [22], we identify a translationally relevant ERBB2 signal network and possible basis for resistance by overexpression of three previously uncharacterized ERBB2 subpathway genes.

## Methods

### Cell lines and mouse xenografts

Human gastric cancer cells were obtained from the American Type Culture Collection (ATCC, Manassas, VA, USA; http://www.atcc.org/) and the SNU Korean Cell Line Bank (http://cellbank.snu.ac.kr/english/). The study was conducted within 6 months of cell resuscitation, followed by culture in RPMI-1640 (Hyclone, Thermo Fisher Scientific; Rockford, IL, USA) and 10 % fetal bovine serum (Hyclone, Thermo Scientific) at 37 °C in 5 % CO_2_. Short tandem repeat (STR) profiling was used to authenticate identity of the cell lines.

For xenograft studies, athymic, 5-week-old male BALB/c nude mice were purchased from Orient Bio Inc. (Gyeonggi, Korea), and kept under specific pathogen-free conditions. Animal experiments were performed under approved protocols and accordance to institutional recommendations for the proper care and use of laboratory animals. To assess in vivo ERBB2 levels, GC cells were suspended in PBS at a concentration of 5x10^7^ cells/ml, and 100-μl inoculum volumes were injected subcutaneously into each mouse’s left and right flanks. The engrafted mice were then observed for four weeks or until subcutaneous tumors became evident.

The usage of animals in this study was reviewed and approved (Project #: NCC-12-R160) by the ethics committee of the National Cancer Center Institutional Review Board (IRB) in accordance with the institute’s rules and regulations. 

### Construction of cell and xenograft microarrays

For the construction of cell microarrays, 5 × 10^6^ cells were pelleted and resuspended in 1 cc of 0.006 % ethel-2-cyanoacrylate containing acetone (Henkel Loctite 401 Super Glue, Henkel, Düsseldorf, Germany), and 5 to 10 volumes 3 % of PVA (Sigma Chemical Co., St. Louis, MO, USA), added to new microcentrifuge tubes, and recentrifuged. The final cell pellets were then wrapped with lens paper and embedded with paraffin to build blocks [23].

### Western blot

Cells were washed with ice-cold phosphate-buffered saline (PBS), scraped from culture flasks, and collected by centrifugation at 2,000 x g. The cell pellets were then resuspended at 1×10^6^ cells in 100-μl lysis buffer (50 mM Tris-HCl, 150 mM NaCl, 2 mM EDTA, 0.5 % NP40, 1 % Triton-X100) with protease and phosphatase inhibitor cocktails (Thermo Fisher Scientific). Cell lysates were kept on ice for 10 min and then centrifuged at 15,000 x g for 15 min at 4 °C. Supernatants were collected and protein concentrations determined by the *DC* protein assay (Bio-Rad, Hercules, CA, USA). 20 μg of total cellular protein was then resolved by SDS-PAGE and transferred to a PVDF membrane (Bio-Rad). After blocking with Tris-buffered saline containing 0.05 % Tween 20 (TBST) and 5 % nonfat milk for 1 h, the membranes were incubated with antibodies against ERRB2 (Abgent, San Diego, CA, USA) and β-actin (Cell Signaling Technology, Beverly, MA, USA) in TBST at 4 °C overnight, and then washed three times with TBST. The washed membranes were then probed with horseradish peroxidase-conjugated anti-rabbit IgG at 1:3000 (Cell Signaling) for 1 h at room temperature, and washed again with TBST. Proteins were visualized by chemiluminescence using the ECL reagent (GE Healthcare, Little Chalfont, UK), and data analyzed using Image Lab (Bio-Rad) software.

### IHC, FISH and SISH of cell lines and xenograft microarrays

Immunohistochemical (IHC) staining was performed on 4-μm tissue sections from paraffin-embedded tissue blocks using the automated staining instrument BenchMark XT and an *i*VIEW DAB Detection Kit with the PATHWAY ERBB2/HER-2/neu (*4B5*) antibody (Ventana Medical Systems, Tucson, AZ, USA), according to the manufacturer’s protocol.

Fluorescent *in situ* hybridization (FISH) was performed on 2-μm tissue sections from paraffin-embedded tissue blocks. Upon xylene deparaffination, antigens were retrieved using TT Mega Milestone (ESBG Scientific, Markham, Ontario, Canada) with CC2 (Cell Conditioning Solution 2, Ventana). Digestion was then performed for 45 min at RT with Pepsin Solution (Kreatech, Inc., Durham, NC, USA). The slides were then washed, dehydrated with ethanol, and air-dried. The PathVysion Kit (PathVysion Her-2 DNA Probe Kit; Abbott, Abbott Park, IL, USA) was then used for in situ hybridization, and DAPI II Counterstain (Abbott) was used for staining nuclei.

Silver in situ hybridization (SISH) was performed on 4-μm tissue sections from paraffin-embedded tissue blocks using an *ultra*View SISH DNP Detection Kit and INFORM ERBB2/HER2 Dual ISH DNA Probe Cocktail, and an automated IHC/ISH slide-staining system, Benchmark XT (Ventana).

### Computational construction of an ERBB2-downstream network from *ERBB2* high- and Low-expressing GC tumor transcriptome datasets, and analysis for genetic anomalies within that network

Using TCGA gastric cancer RNA-Seq datasets retrieved from the UCSC cancer genomics browser (version TCGA_STAD_exp_HiSeq-2015-01-28) [24], a total of 470 cancer samples with pathologic M stage M0 were selected and split into two groups, according to *ERBB2* expression: (1) an *ERBB2*-high expressing sample group (highest 25^th^ percentile); and (2) an *ERBB2*-low expressing sample group (lowest 25^th^ percentile). Each group consisted of 83 samples. We then applied our established systems biology algorithm, PATHOME [22], using a p-value cutoff of 0.05, to distinguish statistically significant RNA-Seq expression data results and delineate signaling networks for the ERBB2 high- vs. low-expressing GC tumor groups. From the network, we selected ERBB2-downstream signaling genes (51 genes, including *ERBB2* itself) and their possible anomalies (using cBioPortal).

### Immunohistochemical (IHC) and fluorescence In situ hybridization (FISH) staining and grading

IHC staining was performed using the BenchMark XT automated staining instrument (Ventana) as follows: formalin-fixed, paraffin-embedded tissue blocks were sectioned at a thickness of 3 μm. The sections were then deparaffinized and rehydrated with EZ prep (Ventana) and washed with Tris-buffered saline. The antigens were retrieved by heat treatment for 30 min in pH 8.0 Tris-EDTA buffer (CC1, Ventana) at 95 °C. Endogenous peroxidases were blocked with 3 % H_2_O_2_ for 10 min at RT. Nonspecific binding was blocked using a ready-to-use protein blocker solution (Ventana) for 20 min at RT. A primary antibody against ERRB2 (1:1000, rabbit polyclonal, A0485, DAKO, Glostrup, Denmark) was then applied to the slide section for 40 min at 42 °C, followed by HRP-labeled secondary Ab for 20 min at RT, and DAB for 8 min (I-View DAB, LSAB, Ventana), with hematoxylin counterstain.

ERBB2 immunostaining was evaluated according to the criteria of Hoffman et al. [25]. Staining was from 0 to 3, as follows: 0, no reactivity or membranous reactivity in <10 % of cells, as follows: 1+, faint membranous reactivity in >10 % of cells; 2+, weak to moderate complete or basolateral membranous reactivity in >10 % of cells; and 3+, moderate to strong complete or basolateral membranous reactivity in >10 % of cells. Biopsy samples with cohesive either IHC3^+^ or ISH^+^ clones were considered positive irrespective of proportion (i.e., <10 %). In cases of IHC2+ staining, FISH for *ERBB* was then performed.

FISH was performed according to the manufacturer’s protocols using the automated staining instrument BenchMark XT (Ventana). Mean HER2:CEP17 ratios were then calculated after 20 tumor cells were counted. The FISH result was considered amplification-present when the HER2:CEP17 ratio was ≥2.0 [18], and negative when the HER2:CEP17 ratio was <1.8 [26]. If the ratio was between 1.8 and 2.0, counting was repeated on an additional 20 tumor cells.

## Results

### Characteristics of a 220-GC tumor cohort and ERBB2-positive cell lines

While ERBB2 expression is well correlated with poor breast cancer prognosis [27, 28], similar studies in gastric cancer (GC) have been inconsistent [29, 30]. However, while it is now approved for GC in many nations, a phase III trastuzumab trial revealed only a modest clinical benefit for ERBB2-positive GC patients [20]. While later studies showed *ERBB2* gene amplification to be fairly homogenous in GC tumors [31], its protein expression levels were much more variable [29, 30].

**Fig. 1:**
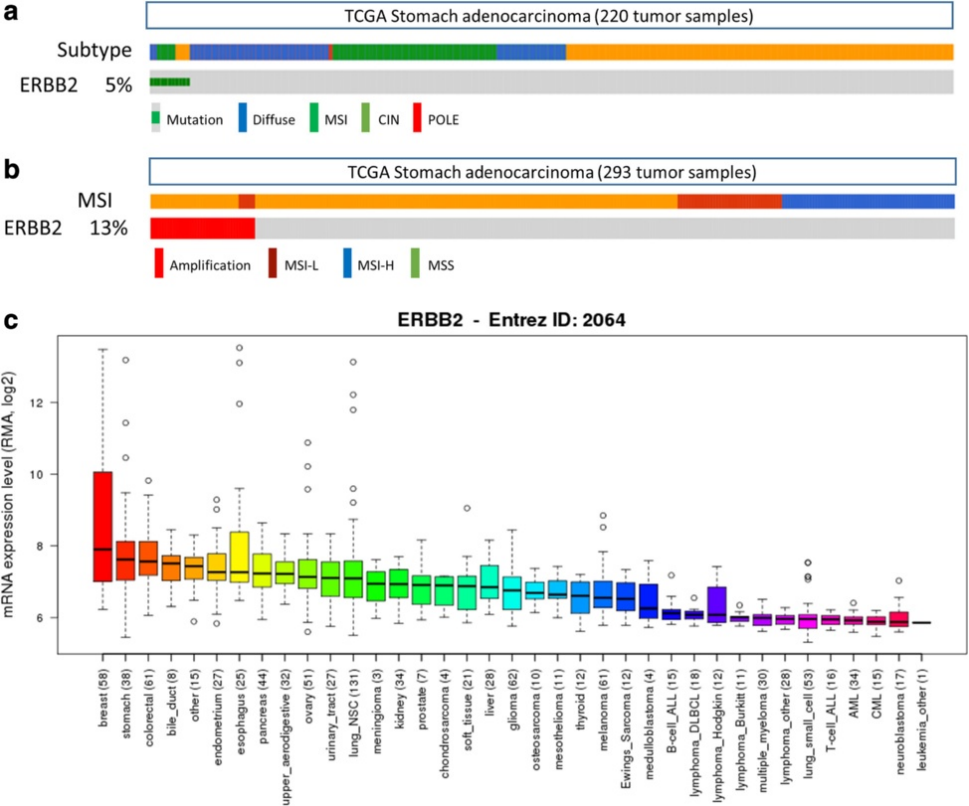
*ERBB2* gene mutation, amplification, and expression in tumors and cell lines. **a** *ERBB2* mutations were observed in 11 (5 %) out of 220 patients. **b** Out of 293 GC patients, 38 (13 %) had ERBB2 amplifications, but not deletions. Interestingly, 38 patients did not have MSI-H status. **a** and **b** used The Cancer Genome Atlas (TCGA: http://cancergenome.nih.gov/). **c** Expression of the *ERBB2* gene throughout various cancer cell lines. We used the Cancer Cell Line Encyclopedia (CCLE). The X-axis represents origins of the cancer cells, and the numeric followed by the origin indicates the number of cell lines assigned to that origin (www.broadinstitute.org/ccle)

Consequently, we assessed *ERBB2* amplification/mutation in 25 highly diverse, ERBB2-positive GC cell lines useful for further trastuzumab study (see below). Examination of a dataset (2014-Jan-12) from The Cancer Genome Atlas (TCGA) [32], through cBioPortal (cbioportal.org) [33], revealed that of 220 tumor samples, a 5 % ERBB2 mutation rate in diffuse, microsatellite-instable (MSI) and chromosome-instable (CIN) gastric cancer subtypes (Fig. 1a), consistent with previous studies [34]. In the second dataset (2014-Jan-28), we found *ERBB2* amplification in 13 % of 293 samples, entirely within tumors having low (<30 %) rates of microsatellite-instability (Fig. 1b), thus showing *ERBB2* copy number to negatively correlate with the presence of repetitive elements. This data represents typical *ERBB2* genomic anomalies seen in gastric cancer [34], which may or may not result in its protein over- or underexpression (see the preclinical tumor models section below).

To further explore the existence and diversity of ERBB2-positive GC cell lines, using the Cancer Cell Line Encyclopedia (CCLE) [35], we found that among 37 tumor types, gastric cancer ranked second in ERBB2 mRNA expression (superseded only by breast cancer, Fig. 1c). Of GC cell lines, as expected, we found that NCI-N87, the basis for most preclinical anti-ERBB2 studies, most highly expressed ERBB2 (Additional file 1: Figure S1). *ERBB2* mRNA expression levels revealed overexpression in eight out of 38 GC cell lines in the CCLE (21.1 %), with quite high standard deviations, similar to the 25-GC cell line panel used in our study.

### Establishment of a diverse GC cell line panel and improved methodologies for inter and intra-sample assessments of ERBB2 protein expression and gene amplification

To model GC patient genotype/phenotype differences (*e.g.*, disease subtype, ERBB2 gene amplification vs. protein overexpression, *etc.*), prior to selection for trastuzumab therapy, we established a panel of 25 highly heterogeneous GC cell lines, using 8 of 14 cell lines from the CCLE, and 17 other cell lines we identified from published literature and public depositories (Additional file 2: Table S1). As expected, NCI-N87 cells showed the highest levels of *ERBB2* gene amplification and protein overexpression (Additional file 1: Figure S1) [36]. Only six of the cell lines had the same Lauren classification [37] as their original tumors (the phase III trastuzumab study demonstrated the diffuse subtype to be the most responsive) [20]. This cell number is still a small number compared to information from the CCLE [35] and patient samples (Fig. 1c). The panel also included several SNU (Seoul National University) GC cells, each with some type of genomic aberration (including single-nucleotide variants, ERBB2 gene amplification, etc.), the well-known GC cell line SNU-216, having both *ERBB2* gene amplification and protein overexpression [18], the cell line NCC-24, originated from an Epstein-Barr virus infection [38], and several other highly distinctive lines (e.g., the MKN series derived from Japanese patient GC metastases) [39]. As many of the cells were derived from Asian GC patient tumors, we also strongly assume that many originated from *H. Pylori* infection [40]. Moreover, similar to breast cancer, we believe this unique, diverse GC line panel reflects the high heterogeneity of GC tumors. Based on others’ convincing assertions that cell lines can accurately reflect the genomic and transcriptomic anomalies of specific GC subtypes [41, 42], we hypothesize that study of the effects of targeted therapeutics on distinct signaling networks (e.g., our independently developed “subpathway” approach) [22], in addition to the identification of possible mechanisms of resistance and drug response-predictive biomarkers, can strongly facilitate the process of translational drug discovery.

### Improved methodologies for rapid and simple determination of ERBB2 protein expression and gene amplification

As discussed above, the targeted drug discovery process begins with study of the effects of the compound of interest on in vitro (cell lines) and in vivo (xenografts) preclinical models of specific cancers. For targeted therapies, it is often desirable to examine the target gene’s copy number, in association with its protein expression levels, and it has been indicated that trastuzumab treatment of gastric cancer is more effective when *ERBB2* gene amplification correlates with high ERBB2 protein expression [30]. Consequently, rapid and straightforward methodologies for examining gene copy number variation and protein expression are highly desirable. Measurement of gene amplification is typically performed by fluorescence in situ hybridization (FISH) and silver in situ hybridization (SISH), while biopsy target protein expression is typically determined using immunohistochemistry (IHC) and/or Western blot. To those ends, we found that for examining cell lines, the cell microarray (CMA) is a more physiologically relevant method (i.e., 3-dimensional growth), compared to mere monolayer growth on plates pre-coated with poly-lysine or various extracellular matrix proteins. The CMA is an array of cells fixed into blocks from which a round slice of the sample is placed on a glass slide. The sample can subsequently be used for IHC of the desired protein of interest or FISH/SISH determinations of gene copy number. CMA also eliminates potential cell variability due to passage number and contamination [43].

**Fig. 2:**
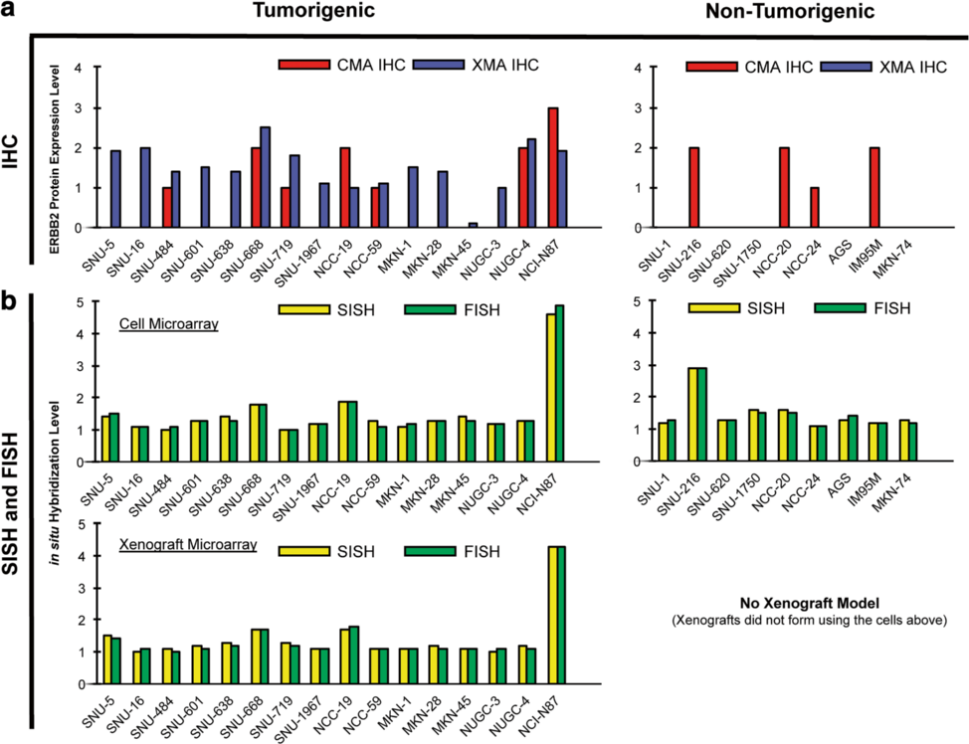
Assessment of concordance of in vitro ERBB2 protein levels. **a** ERBB2 protein expression, as assessed by immunochemistry (IHC) of cell lines (red bars) or xenografts (blue bars) **b** Assessment of *ERBB2* copy number by fluorescence *in situ* hybridization (FISH, green bars) or silver *in situ* hybridization (SISH, yellow bars) in cell lines (upper panel) or xenografts (lower panel)

Similarly, for rapid and straightforward examination of in vivo gene copy number and protein expression, the xenograft microarray (XMA) is an array of tumor tissue samples made after injecting and growing cancer cells subcutaneously in nude mice [44]. Once the cells comprise a tumor of the desired size, tumor is dissected out and used for the construction of a microarray. In this study, both CMA and XMA were developed to rapidly and accurately determine ERBB2 protein expression (IHC) and gene amplification (FISH and SISH) (Fig. 2).

### Discordance between in vitro and in vivo ERBB2 protein levels in GC cell lines

We next assessed the extent of correlation between protein (IHC, Fig. 2a) and gene copy number (SISH and FISH, Fig. 2b). With the exception of NCI-N87 GC cells (>4.0), we observed fairly similar low levels of ERBB2 gene expression level (~1.0–1.5).

Of likely greater clinical relevance, we assessed whether ERBB2 gene/protein expression levels were reproducible between in vitro (cell culture) and in vivo (mouse xenograft) conditions. As shown in Fig. 2a (blue bars), eleven of the 25 GC cell lines showed ERBB2 expression, as determined by IHC of CMA. Remarkably, however, merely 16 of the lines were tumorigenic and expressed ERBB2 protein in vivo (as determined by IHC of XMAs), including eight lines in which protein expression was completely absent in vitro (CMAs) (Fig. 2a, left panel). One GC line, MKN-45, formed tumors while ERBB2 expression was barely detectable. Also curiously, three cell lines (SNU-16, NCC-20, and IM95M) with fairly high ERBB2 expression in vitro, entirely failed to grow tumors (Fig. 2a, right panel).

**Fig. 3:**
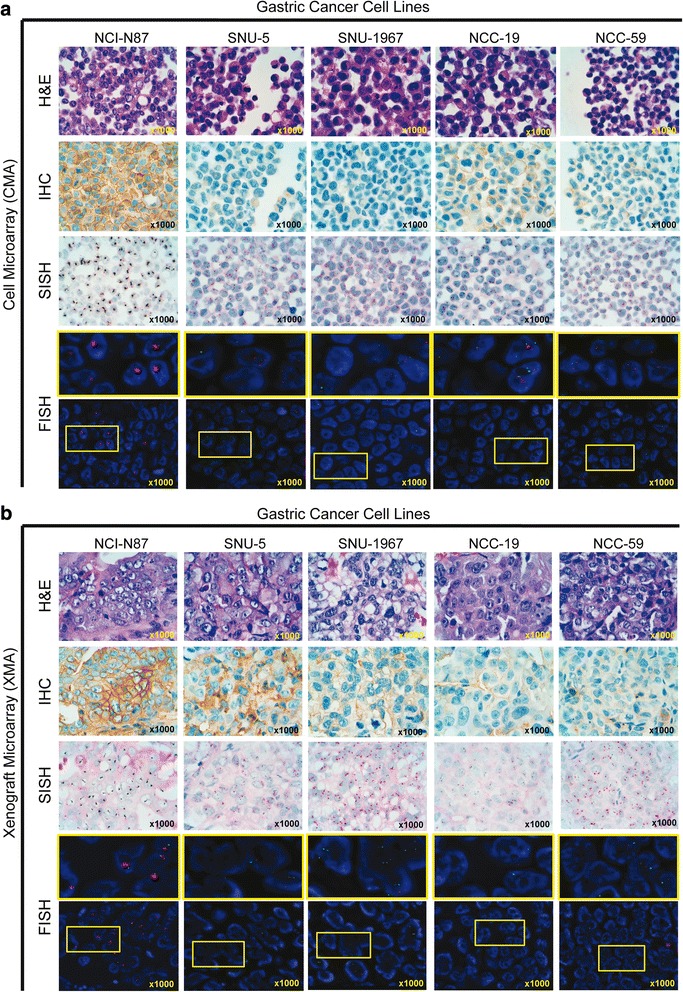
Assessment of concordance of *in vivo* ERBB2 proteins levels in five distinct gastric cancer cell lines, as determined by H&E (hematoxylin and eosin stain, left panel) and immunohistochemistry (IHC, middle panel) and ERBB2 gene expression levels, as determined by silver *in situ* hybridization (SISH, right panel), using five separate cell (CMAs) **a** or xenograft **b** microarrays (XMAs)

Similarly, with the exception of NCI-N87 cells, we also noted a discordance of gene amplification (low) and protein expression (high) in SNU5 GC cells, using CMAs (Fig. 3a) and XMAs (Fig. 3b), while 5/6 cell lines we examined in more depth showed highly heterogeneous ERBB2 protein expression (Fig. 3a – b). Of the total 15/25 tumorigenic lines overall, expression levels were quite heterogeneous (0.1 – 3), similar to findings in human GC tumors [20, 29]. Future “omics” analysis of this cell line panel will allow us to determine their degrees of similarity with specific disease subtypes. We further believe that drug testing on diverse cell lines might better represent the many heterogeneous cell types present in specific human (e.g., gastric, pancreatic, etc.) tumors [30]. These results also demonstrate the strong contribution of the tumor microenvironment to growth of tumor xenografts, including its in vivo influence on implanted cancer cells.

### Assessment of correlation between ERBB2 expression and GC stage in Asian vs. Caucasian patient tumor cohorts

While tumor *ERBB2* overexpression/gene amplification correlates with poor prognosis in breast cancer [17, 26, 27], similar studies in gastric cancer have been inconsistent [29, 45]. Thus, we assessed the prognostic value of *ERBB2* expression with respect to GC disease grade, in Asian (GEO accession: GSE36968) [46] vs. Caucasian (GEO accession: GSE63288) [47] patient tumors. For determining statistically relevant associations, we used one-way ANOVA and Tukey’s tests. As shown in Additional file 3: Table S2 and Additional file 4: Figure S2A, the Asian dataset showed no significant association between disease stage and *ERBB2* expression. The Caucasian dataset, however, did show an odd pattern of stage differences associated with *ERBB2* expression, which further varied by statistical method. For example, using one-way ANOVA, disease stages I, II, and III could each be distinguished from normal stomach by ERBB2 positivity; using Tukey’s test, however, only stage II could be differentiated from normal tissue, and stage III from stage II, based on *ERBB2* expression (Additional file 4: Figure S2B and Additional file 5: Table S3). These results again underscore the inconsistent prognostic value of ERBB2 for GC, and our additional finding that Asian GC patients lacked any stage relatedness to *ERBB2* expression might indicate additional race-related ERBB2 cofactors involved in GC etiology, based on the higher disease incidence in that population compared to Caucasians.

### Computational construction of an ERBB2 downstream network from *ERBB2* high- and low-expressing GC datasets, and analysis for genetic anomalies within that network

**Fig. 4:**
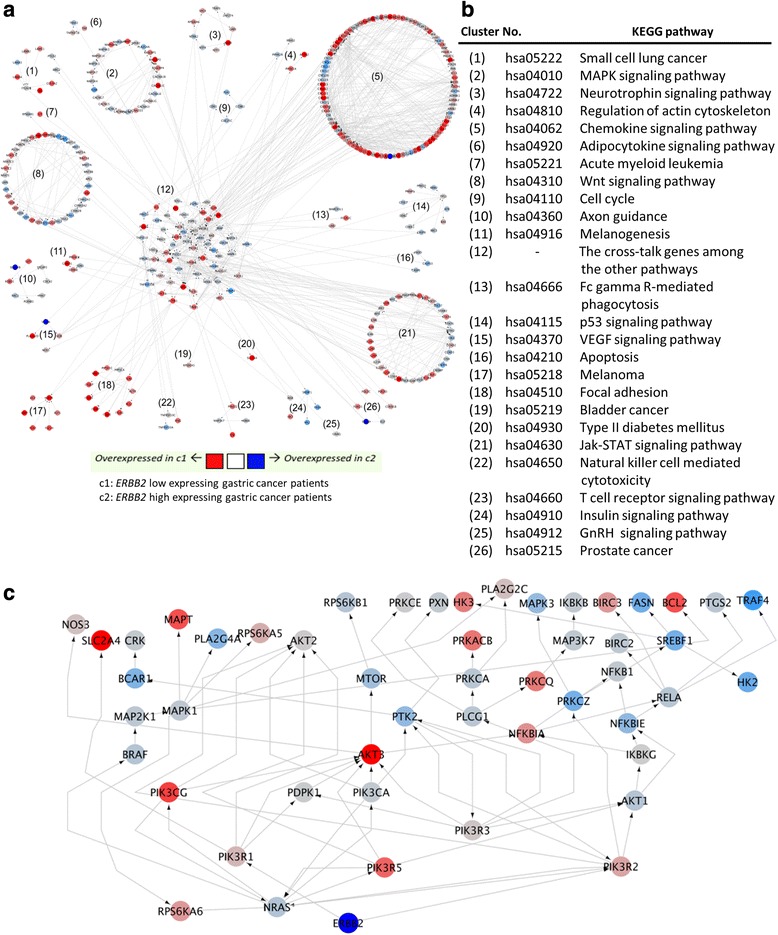
Signaling network is common to both *ERBB2* high- and low-expressing gastric cancer patients in TCGA. **a** The network was delineated by our established systems biology algorithm, PATHOME [22]. The network consists of subsets of multiple KEGG pathways, as indicated by the numerals. Nodes represent gene symbols, with the depth of red indicating greater upregulation in the *ERBB2* low-expressing patient group. The depth of the blue color indicates upregulation in *ERBB2* high-expressing patients group. **b** KEGG information provided according to the numerals in **a. c** ERBB2 downstream signaling. We extracted ERBB2 downstream from Fig. 4a, revealing 51 downstream genes (including *ERBB2* itself)

Finally, we examined *ERBB2* expression in GC tumor RNA-seq datasets from The Cancer Genome Atlas (TCGA) [32]. We divided the GC tumor datasets into two groups according to high vs. low *ERBB2* expression (see details in the Materials and Methods). To understand signaling common to both *ERBB2* high- and low-expressing GC patients, we employed our established systems biology algorithm [22], resulting in an ERBB2-centric network (Fig. 4a). As shown, we found 26 KEGG pathways associated with ERBB2 high- *vs*. low-expressing patients; these are listed in Fig. 4b. We also observed that within the network, individual pathways significantly cross-talked with one another (see cluster (12) in Fig. 4a).

**Fig. 5:**
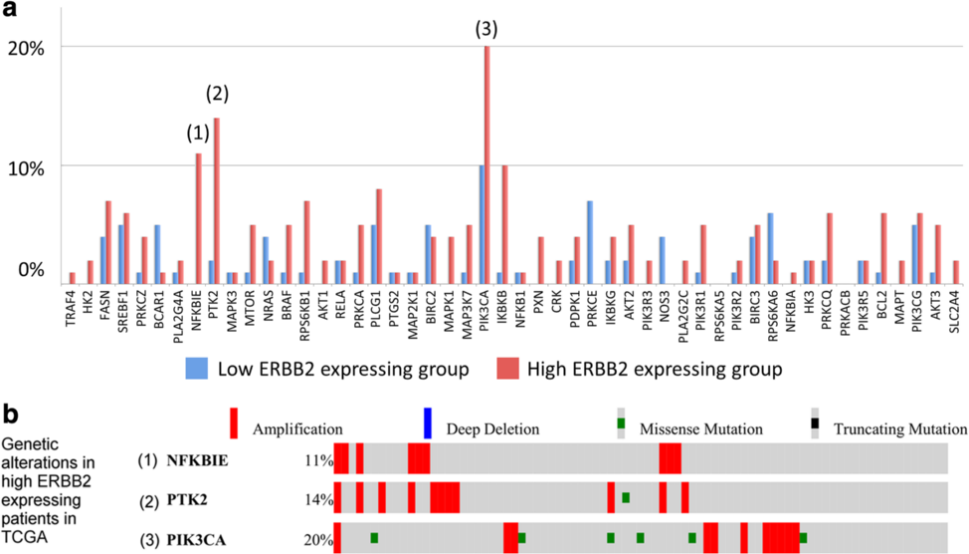
Identification of genetic alterations of *ERBB2* downstream signaling genes between the high- vs. low-expressing groups. **a** From the *ERBB2* downstream genes (including *ERBB2* itself), we identified genetic alterations (e.g., copy number variations, mutations) between the two GC patient groups using cBioPortal (cbioportal.org) at its default setting. The y-axis represents the percentage of patients with the altered gene, in terms of copy number variations and mutations. The red bar indicates the high *ERBB2*-expressing sample group, and the blue bar indicates the low *ERBB2*-expressing sample group. The three genes (*PIK3CA, PTK2, NFKBIE*), indicated by numbers showed the most alteration in the high *ERBB2*-expressing group, compared to the low *ERBB2*-expressing group. **b** The genetic alteration profile for the three genes (*PIK3CA, PTK2*, and *NFKBIE*) indicated in the numbers above, is shown for the high *ERBB2*-expressing GC patients

From the network (Fig. 4b), we further dissected how ERBB2 transduced its signal into its downstream network (Fig. 4c), and how trastuzumab might target ERBB2-relating signaling. The ERBB2 downstream network consisted of 51 genes, from which we identified genetic alterations using CBioPortal (cbioportal.org). Three genes, *PIK3CA*, *PTK2*, and *NFKBIE*, positively correlated with *ERBB2* expression (Fig. 5a), while Fig. 5b shows each of these genes to harbor large regions of amplification. Our analysis strongly suggests that another cascade, consisting of highly amplified *PTK2* and *NFKBIE*, could be strongly involved in ERBB2-related signaling in high *ERRB2*-expressing, trastuzumab GC patients. To further validate these computational results, we will further examine our 25-cell line panel for *PTK2* and *NFKBIE* aberrations, both in vitro and in vivo.

## Discussion

In this study, we endeavored to extensively characterize the potential of ERBB2 as a clinical biomarker through a myriad of in vitro and in vivo diverse gastric cancer (GC) models and human tumors, with an overall objective of replicating GC tumor heterogeneity and identifying pathways/networks that could contribute to trastuzumab resistance.

Similar to the earliest, preclinical discovery phases for all antineoplastic therapies, the efficacy of trastuzumab for GC was based on cell line studies. These studies, however, were largely performed on a single cell line, NCI-N87, in which the *ERBB2* gene is highly amplified, and its protein product is expressed at very high levels [18, 19]. While gene amplification was found relatively constant in ERBB2-positive GC tumors [31], its protein expression was highly heterogeneous, often, even within the same tumor [22]. This finding might suggest that a significant number of GC patient tumors are intrinsically resistant to trastuzumab, but this has yet to be statistically or analytically validated [48, 49].

While for some cancers, intrinsic drug resistance to targeted therapies may be modest or negligible (e.g., breast cancer response to trastuzumab), most cancers eventually gain *acquired* resistance to targeted therapies [1]. Acquired resistance can arise due to numerous factors, including somatic mutations, downregulation of the therapeutic target, intratumoral heterogeneity, epigenetic aberrations, and a shift of cancer cell reliance to alternative or target-downstream mitogenic signaling pathways. Indeed, our data analysis of ERBB2-positive tumors in The Cancer Genome Atlas revealed amplification and/or missense mutations in the ERBB2 signal pathway downstream genes *NFKBIE*, *PTK2*, and *PIK3CA*. Being downstream of, and thus bypassing, the therapeutic target, represents one mechanism of cell survival that can lead to drug resistance and tumor relapse, possibly requiring additional therapies. For example, a recent report showing highly aggressive (“triple negative”) breast cancer was vulnerable to double inhibition of HER2/mTORC1 (HER2 by trastuzumab and mTORC1 by a selective inhibitor, INK-128) [50]. Other dual-therapy regimens being examined are combined inhibition of AKT/mTOR and MDM2 in glioblastoma [51], and combined c-MET and EGFR in non-small lung cancer [52]. With further regard to HER2 in breast cancer, it was found that the EGFR inhibitor gefitinib did not enhance the anti-cancer activity of trastuzumab when used alone [53].

While disappointing clinical trials often arise from preclinical studies of inadequate quality, *improved in vitro* and in vivo cell line models must continue to serve as a basis for the first step (i.e., screening) of discovery of candidate anti-cancer compounds, combined with rigorous network analysis of the potential therapeutic target. Moreover, various guidelines have been offered to improve candidate drug screening using cell lines and tumor xenografts [3]. Recent “omics” profiling can also facilitate cell lines that best represent their respective tumors, similar to recent studies identifying distinct breast cancer cell lines with genomic profiles (including copy number alterations) that represent specific breast cancer disease subtypes [41, 42].

In summary, we assert that drug screening should be performed using multiple, diverse cell lines from the same tumor type, particularly for highly heterogeneous tumors (i.e., gastric or pancreatic tumors). Combining this approach, with bioinformatics analyses of subtype-specific tumors (followed by cell line validation of the computational results) could even further improve preclinical drug screening (and possible mechanisms of therapy resistance), and aid in the discovery of predictive response biomarkers, as has been asserted by others. Integration of all these approaches will improve success rates in “personalizing” particular therapies to patients who are most likely to benefit.

## Conclusions

“Personalized medicine,” or tailoring therapies to specific individuals based on their expression of various biomarkers, has had some amount of success. However, highly aggressive cancers might fail to respond to the therapy at all (innate recalcitrance) or acquire resistance following treatment, due to clonal selection of tumor cells that downregulate the target, gain activity of downstream or parallel signaling pathways, or are highly heterogeneous. Moreover, while the FDA (and other regulatory bodies) exhaustively vet new drugs, such strict regulation of biomarkers has yet to be established. Consequently, to better design preclinical compound candidates (and biomarker assays) for eventual translation, one must consider all of these factors. We assert that our approach, using highly heterogeneous cancer cell lines, consideration of the behavior of tumor xenografts, and rigorous network analysis to delineate possible signaling “subpathways” that can bypass specific biomarker-driven targets, can achieve many of these elusive goals.

## Additional files

Additional file 1: Figure S1A. Of 38 gastric cancer cell lines, we found that NCI-N87 cells, the basis for most preclinical anti-ERBB2 studies, had the highest ERBB2 expression level. (DOC 152 kb) Additional file 2: Table S1. Characteristics of gastric cancer cell line panel of 25 cell lines. (DOC 116 kb) Additional file 3: Table S2. Stage comparison according to ERBB2 expression in Asian (GEO accession: GSE36968). For comparisons, we used one-way ANOVA and Tukey’s test. In the Asian dataset, there were no significant differences between stages according to ERBB2 expression. (DOC 89 kb) Additional file 4: Figure S2A. According to gastric cancer disease stage, ERBB2 expression in Asian (GEO accession: GSE36968) and Caucasian (GEO accession: GSE63288) datasets. a Box plots depicting ERBB2 expression in the Asian dataset (GEO accession: GSE36968). The x-axis represents stages and the y-axis is the logarithm of RPKM (a measure of gene expression). b Box plots depicting ERBB2 expression in the Caucasian dataset (GEO accession: GSE63288). The dataset did not contain stage IV patients. The x-axis represents GC disease stages and the y-axis is the logarithm of RPKMs. (DOC 88 kb) Additional file 5: Table S3. Stage comparison according to ERBB2 expression in Caucasian (GEO accession: GSE63288). For comparisons, we used one-way ANOVA and Tukey’s test. In the Caucasian dataset, there were significant stage differences according to ERBB2 expression. The significant p-value was in italic. (DOC 75 kb)

## Abbreviations

CMA: cell microarray
ERBB2: epidermal growth factor receptor-2
FISH: fluorescent *in situ* hybridization
SISH: silver *in situ* hybridization
XMA: xenograft microarray
