# Supplementary figures and images for: Improving gastric cancer preclinical studies using diverse in vitro and in vivo model systems
Source: BMC Cancer. 2016 Mar 9;16:200. doi: 10.1186/s12885-016-2232-2 (PMC4784390; doi:10.1186/s12885-016-2232-2)

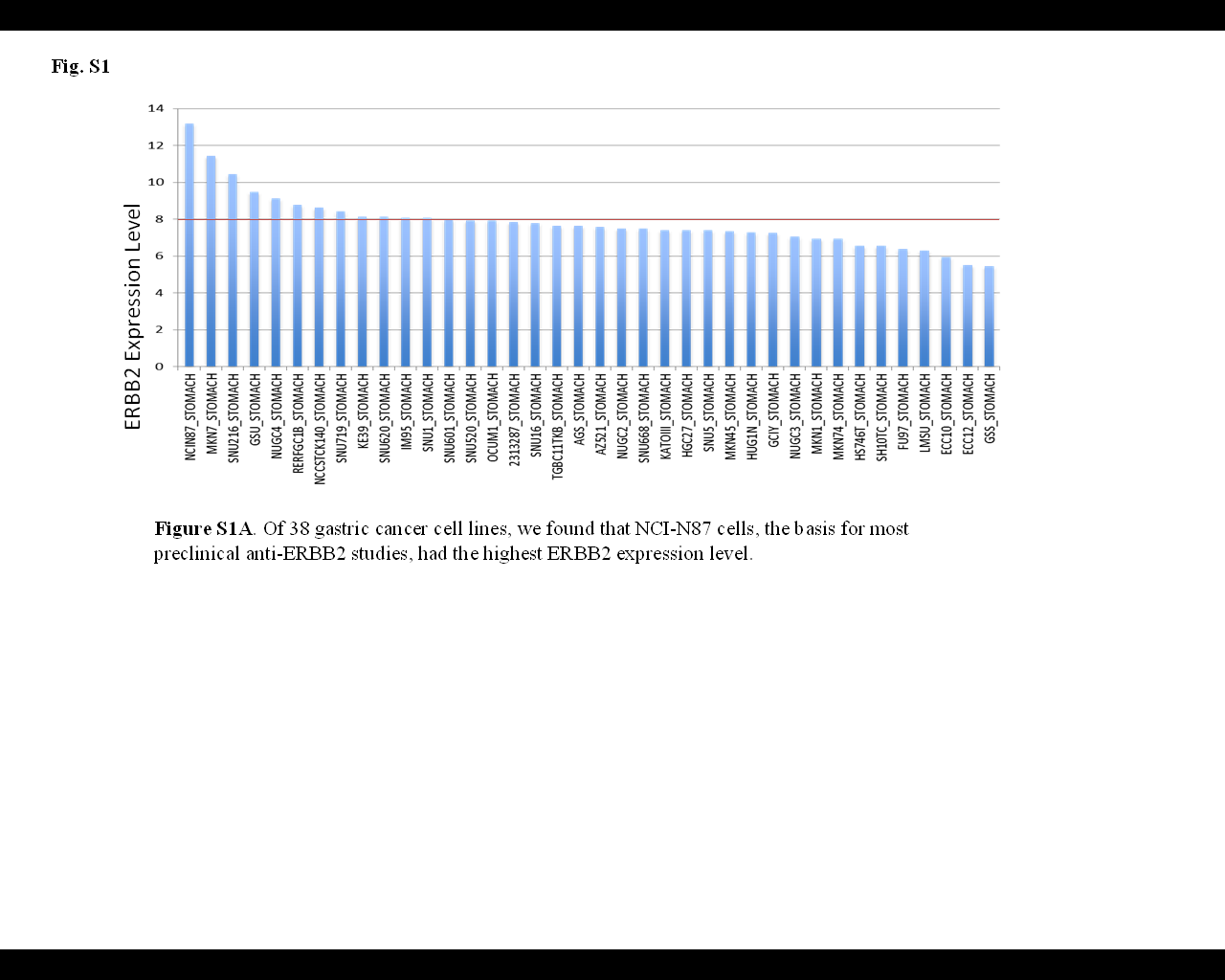

Supplement: Additional file 1: Figure S1A. — Of 38 gastric cancer cell lines, we found that NCI-N87 cells, the basis for most preclinical anti-ERBB2 studies, had the highest ERBB2 expression level. (DOC 152 kb) [file 12885_2016_2232_MOESM1_ESM.doc]

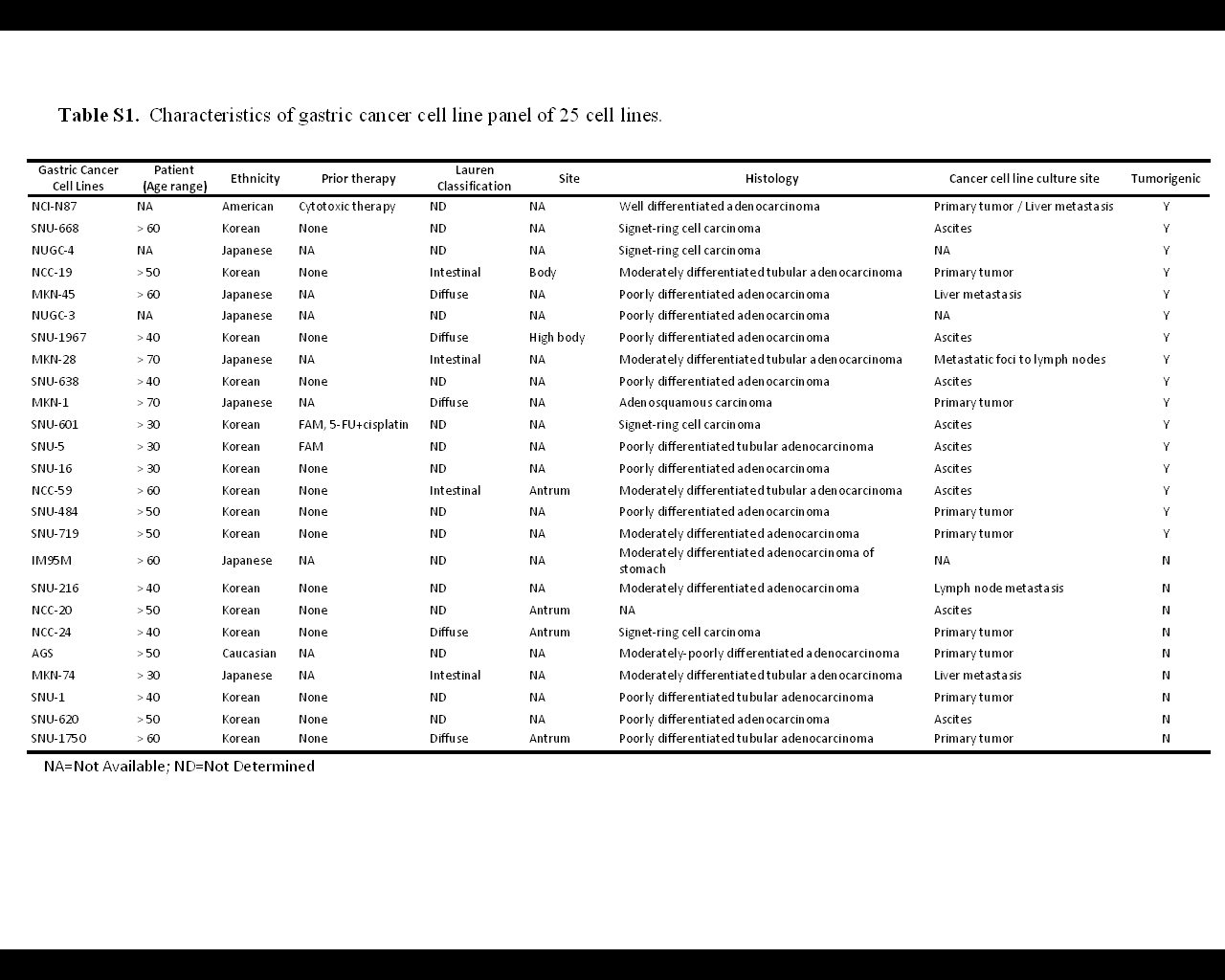

Supplement: Additional file 2: Table S1. — Characteristics of gastric cancer cell line panel of 25 cell lines. (DOC 116 kb) [file 12885_2016_2232_MOESM2_ESM.doc]

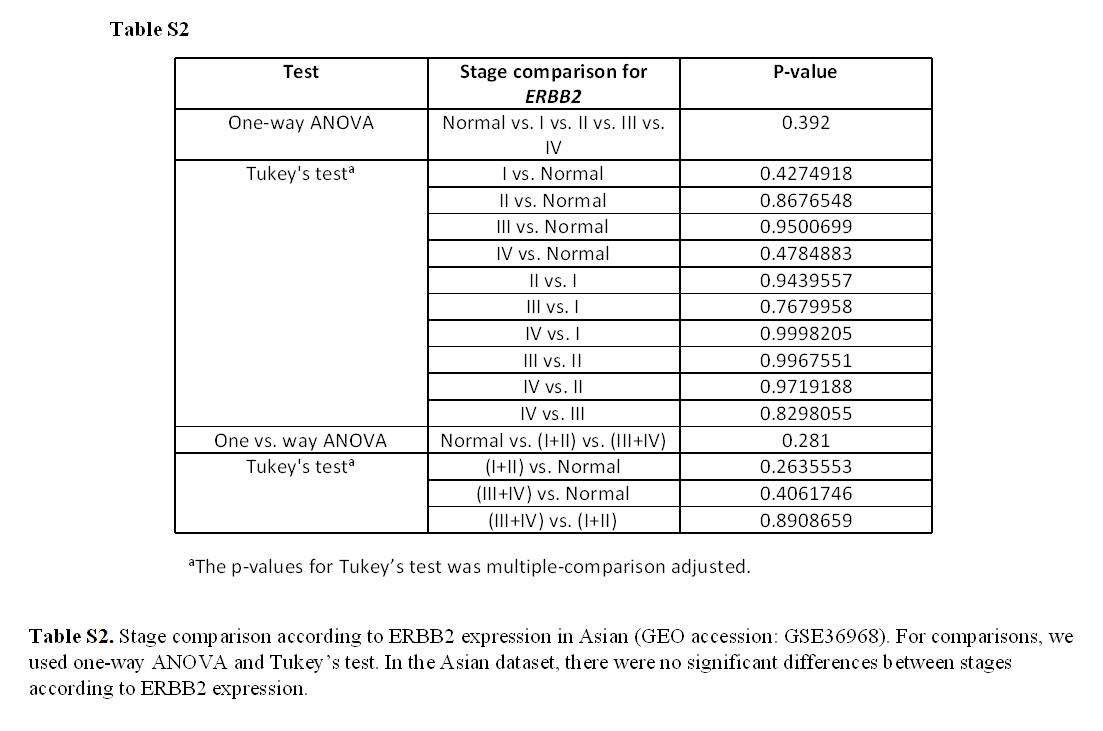

Supplement: Additional file 3: Table S2. — Stage comparison according to ERBB2 expression in Asian (GEO accession: GSE36968). For comparisons, we used one-way ANOVA and Tukey’s test. In the Asian dataset, there were no significant differences between stages according to ERBB2 expression. (DOC 89 kb) [file 12885_2016_2232_MOESM3_ESM.doc]

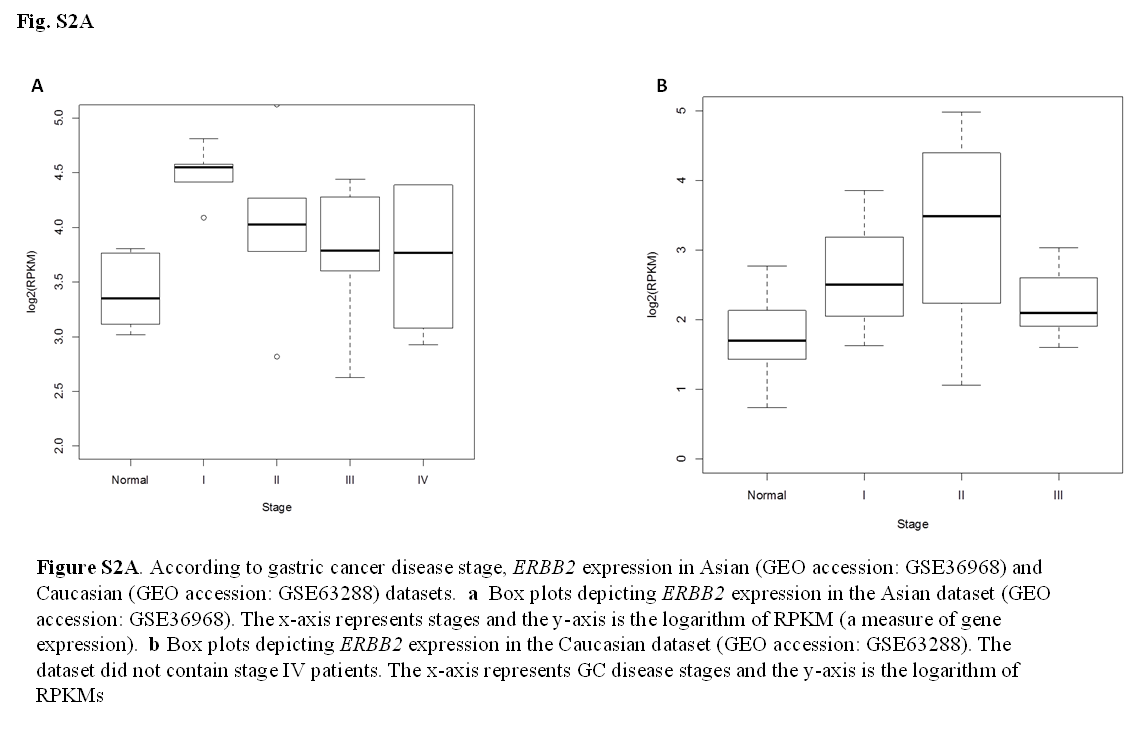

Supplement: Additional file 4: Figure S2A. — According to gastric cancer disease stage, ERBB2 expression in Asian (GEO accession: GSE36968) and Caucasian (GEO accession: GSE63288) datasets. a Box plots depicting ERBB2 expression in the Asian dataset (GEO accession: GSE36968). The x-axis represents stages and the y-axis is the logarithm of RPKM (a measure of gene expression). b Box plots depicting ERBB2 expression in the Caucasian dataset (GEO accession: GSE63288). The dataset did not contain stage IV patients. The x-axis represents GC disease stages and the y-axis is the logarithm of RPKMs. (DOC 88 kb) [file 12885_2016_2232_MOESM4_ESM.doc]

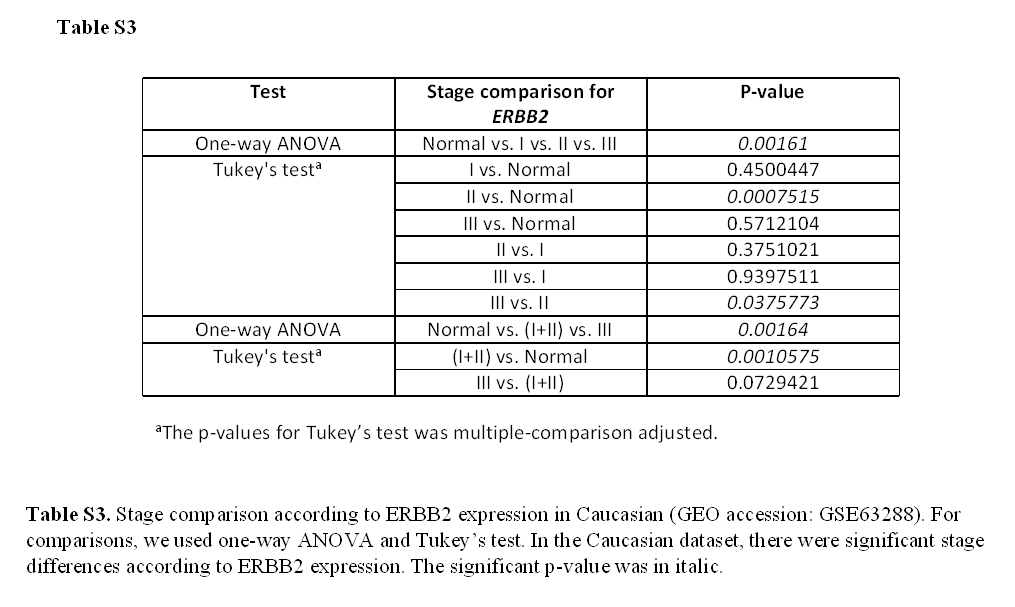

Supplement: Additional file 5: Table S3. — Stage comparison according to ERBB2 expression in Caucasian (GEO accession: GSE63288). For comparisons, we used one-way ANOVA and Tukey’s test. In the Caucasian dataset, there were significant stage differences according to ERBB2 expression. The significant p-value was in italic. (DOC 75 kb) [file 12885_2016_2232_MOESM5_ESM.doc]
